# Femoral neck stress fracture in a young female recruit: case report

**DOI:** 10.1051/sicotj/2018011

**Published:** 2018-05-18

**Authors:** Hélder Fonte, Ricardo Rodrigues-Pinto

**Affiliations:** 1 Portuguese Army Portugal; 2 Centro Hospitalar do Porto, Hospital de Santo António, Largo Professor Abel Salazar, 4099-001 Porto Portugal

**Keywords:** Femoral neck, Stress fracture, Exercise, Military, Female

## Abstract

*Introduction*: Femoral neck stress fractures are uncommon and depending on their location, can be at high risk for non-union and significant morbidity.  Their prevalence is higher among runners and military recruits, and women seem to be at higher risk.

*Methods*: A 27-year-old female, who was enrolled in military recruit, reported left side groin pain after a strenuous running exercise. Due to persistent pain an X-Ray was ordered, which revealed no signs of acute lesions. Further imaging studies with CT scan and MRI identified a compression-type femoral neck stress fracture.

*Results*: The patient was submitted to conservative treatment consisting of restricting from full weight-bearing. Six weeks after she initiated partial weight-bearing, becoming asymptomatic at seven months. Follow-up imaging studies revealed union of the fracture.

*Discussion*: This diagnosis should be considered when evaluating military and athlete populations. Early recognition of these injuries is crucial because complication and morbidity rates are high.

## Introduction

Femoral Neck Stress Fractures (FNSF) are rare, representing 5% of all stress fractures, and most prevalent among runners and military recruits [1]. Women are at higher risk in both populations with an overall incidence of stress fractures of 9.2% (vs. 3% for males) in the military and 9.7% (vs. 6.5% for males) in athletes [2]. The majority of these fractures are undisplaced. Early diagnosis with appropriate imaging in patients with a history of groin pain during training might be crucial in detecting fractures at early stages [1]. The goal of this paper was to report a case of femoral neck stress fracture in a young female recruit and to highlight the importance of early suspicion of this type of injuries in the military and athlete populations, especially in females, providing the right treatment and avoiding possible complications.

## Materials and methods

**Figure 1:**
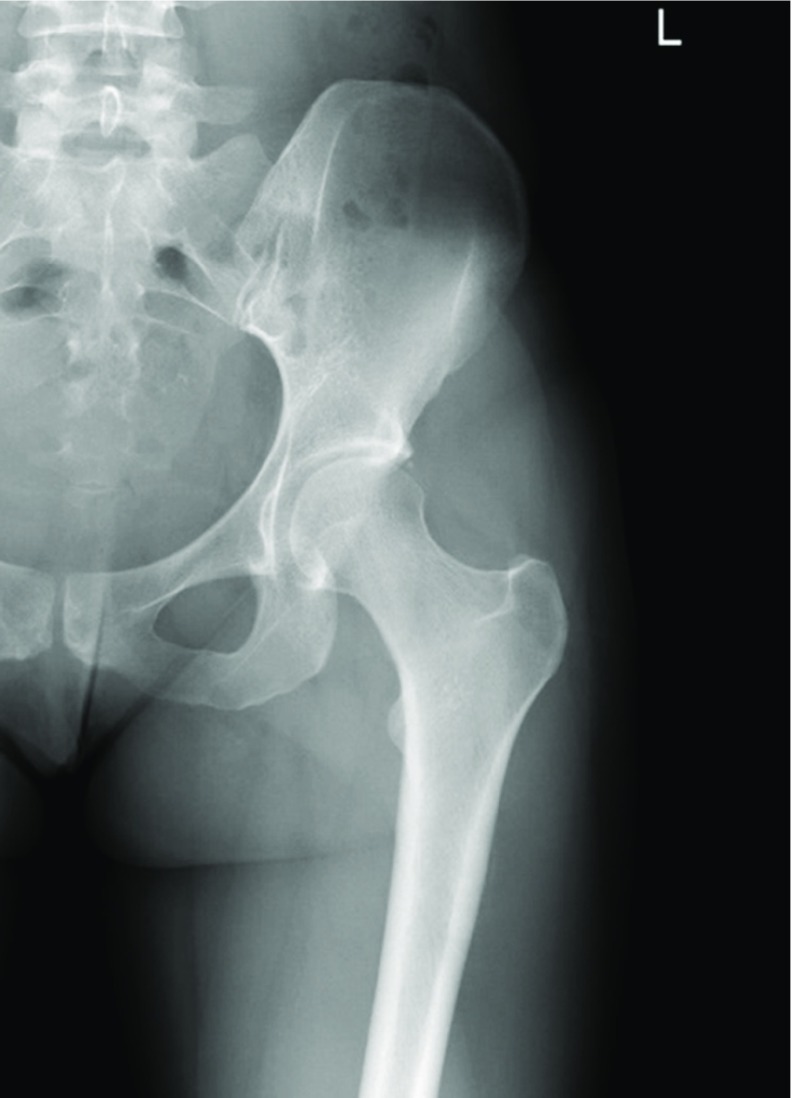
X-ray of the left hip (AP view), performed one month after the onset of the symptoms, which shows no signs of fracture.

**Figure 2:**
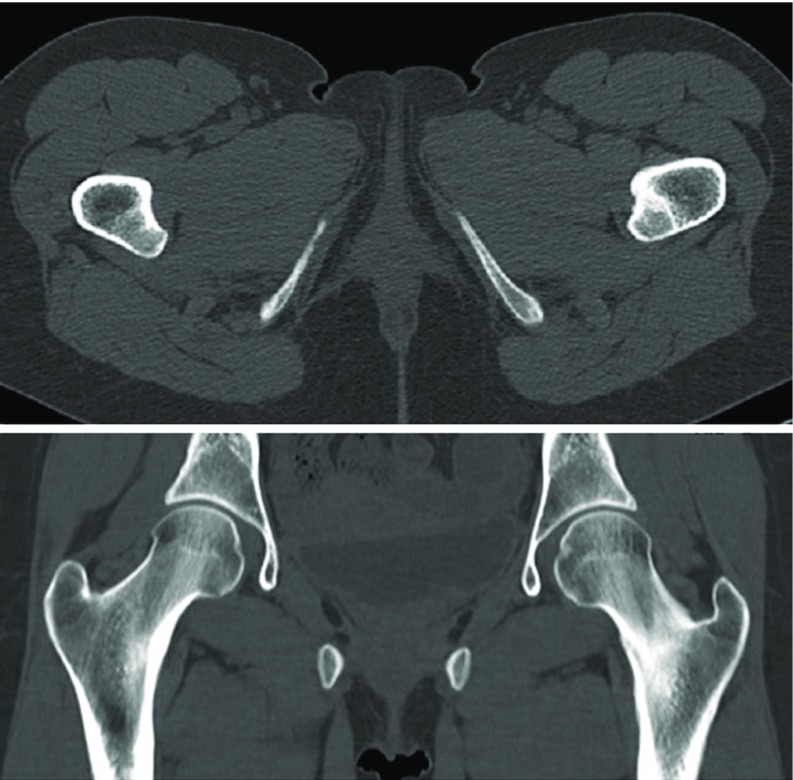
Pelvic CT-scan (A – axial view, B − coronal view) showing disruption of the medial cortex of left femoral neck.

**Figure 3:**
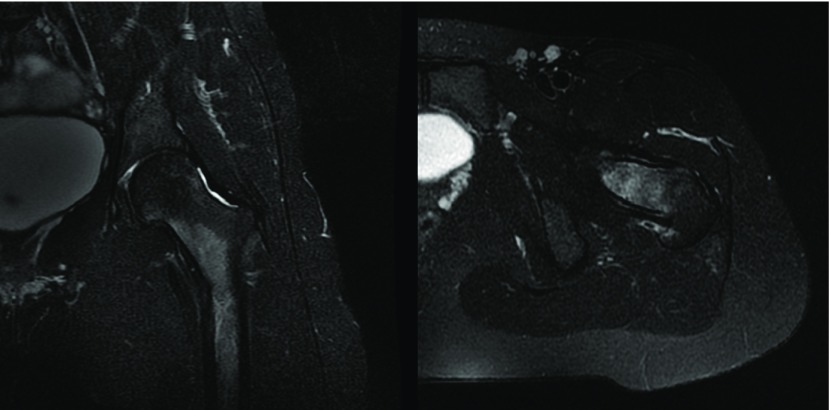
MRI of the left hip, STIR sequence, showing hyperintense signal, characteristic of a compression type femoral neck stress fracture.

A 27-year-old female military recruit reported left side groin pain after a strenuous running exercise, in which she ran 6 km in less than 30 min, wearing a uniform and boots, and carrying a 4.5 kg riffle. After one week taking oral Diclofenac (50 mg bid) and with only partial pain relief, she presented to the military medical facility and was given intramuscular analgesia (diclofenac 75 mg/3 mL and thiocolchicoside 4 mg/2 mL). Three weeks later she returned due to worsening of the symptoms. On physical examination no deformities were noted and the left hip active and passive range of motion were slightly reduced (internal rotation: 35°, external rotation: 45°, extension: 30°, flexion: 120°) with mild discomfort on the extremes of range of motion. The affected limb was well perfused with normal peripheral pulses and normal motor sensory function. The patient reported amenorrhea since the beginning of military training and for two months. A left hip AP X-Ray was ordered but no signs of fracture or other acute lesions were identified (Figure 1). However, as the symptoms persisted for two weeks longer, and despite the medication and rest, a CT scan was done, that revealed a disruption of the medial cortex of the left femoral neck (Figure 2). She was referred to be seen by an Orthopaedic surgeon at the Military Hospital. An MRI (Figure 3) was done that showed a nondisplaced compression-type left femoral neck fracture. The fracture line involved slightly less than 50% of the width of the femoral neck. The patient was advised to a six-week period of non-weight bearing, using crutches and restriction from physical activity.

## Results

**Figure 4:**
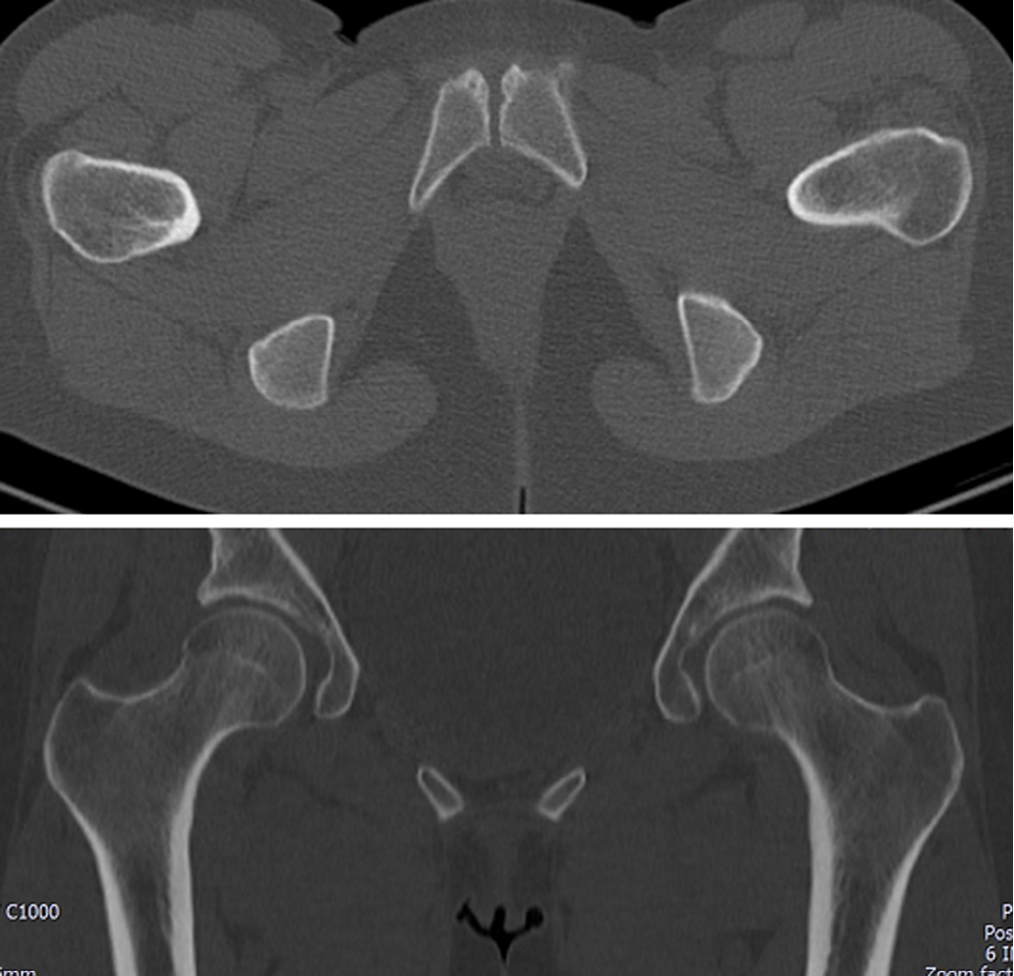
Pelvic CT-scan (A – axial view, B − coronal view) at twelve months, showing fracture union and no signs of complications.

Analytical studies including haemoglobin concentration, iron and vitamin D levels were normal. One month after starting the treatment the menstrual cycles returned. After the initial six weeks, the patient initiated partial weight-bearing and at three months she started light impact activities. Seven months after the beginning of conservative treatment, the patient was asymptomatic. Follow-up imaging studies, including X-rays and a CT scan at seven and twelve months revealed union of the femoral neck fracture and no evidence of complications, such as delayed union or avascular necrosis of the femoral head (Figure 4). The patient gradually returned to full duty exercise activities, such as running, without symptoms. At 18 months the patient remains asymptomatic and has no limitations on her military activities.

### Informed consent

Informed consent was obtained from the patient for publication of this case.

## Discussion

Stress fractures are defined as the mechanical failure of bone due to repetitive loading, which exceeds its structural strength. Failure can occur in a normal bone exposed to abnormal stresses (fatigue fractures) or in abnormal bone that cannot compensate normal stress loading (insufficiency fractures). Stress fractures caused by sports activities predominantly represent the fatigue type and are located almost exclusively in the lower extremity [3].

FNSF are particularly devastating for the career of military recruits as they may imply discharge from military service and a prolonged recovery. Patients typically present with anterior groin pain and inability to bear weight. Tenderness to palpation is not common, but pain with extremes of internal and external rotation may elicit discomfort [4]. Other symptoms, such as an antalgic gait and limited range of motion are also commonly reported and lower limb shortening is typically encountered in displaced fractures [3].

Early radiographs of the hip fail to identify stress fractures in 30–70% of cases. Radiologic evidence of fractures occurs when about 40% of the bone structure is altered and osseous changes become visible − up to 2–3 weeks after the onset of symptoms. [3] Kuhn MK. et al. established a relation between acetabular retroversion and FNSF after reviewing the radiographs of recruits with history of FNSF based on the principle that additional stress caused by abnormal hip mechanics may result in further microdamage [5].  Carey et al. demonstrated that military recruits have a high prevalence of radiographic findings suggestive of femoroacetabular impingement, as could be interpreted in the initial X-ray in this case. MRI may be warranted if symptoms fail to improve and will show bony edema (most commonly in the compression side of the femoral neck), and a fracture line may be present [4].

Establishing the correct diagnosis is often delayed in femoral neck stress fractures. In this case the time between the onset of symptoms and diagnosis was 2 months. Neubauer and colleagues have recently performed a systematic review of 48 stress fractures of the femoral head in runners and found that the mean delay in diagnosis was 57 days [3].

A high degree of suspicion should be present after sudden increases or changes in training volume. At-risk populations include patients with prior stress fractures, patients with medical history predisposing to fracture and females with athlete triad [2]. The latter refers to the interrelationship among energy availability, menstrual function, and bone mineral density, which may have clinical manifestations including eating disorders, functional hypothalamic, amenorrhea and osteoporosis. Runner's highest rates of bone injury may be related to their increased prevalence of female athlete triad, leading to multiple hormonal adaptations that negatively affect the menstrual cycle and bone metabolism. In this case while amenorrhea was present, which in some studies has been proposed as a risk factor *per si*, the complete triad was not present.

Out of various classifications, the systems of Blickenstaff and Fullerton are the most popular. In the latter, fractures are classified according to their location and degree of displacement in compression type (Type I) at the medial cortex of the femoral neck, tension type (Type II) at the lateral cortex or displaced fractures (Type III). In general, compression fractures are considered stable and can be treated conservatively with rest and non-weight-bearing, while tension or displaced fractures need surgical fixation [3]. Displacement of the femoral neck is the main determinant of outcome [6]. Early suspicion and appropriate treatment is important for reducing complications and delayed rehabilitation [4]. FNSF are associated with a prolonged rehabilitation course and extended absence from sports and active military duty.

In conclusion, stress fractures have been described in runners and in the military recruit population. They typically involve the lower extremity [3]. Although relatively rare, unrecognized or untreated FNSF carry a much higher morbidity rate than other stress fractures. It is the author's opinion that athletes and militaries complaining of hip or groin pain after strenuous exercise, especially in at risk populations, as aforementioned, should undergo an x-ray at first evaluation and, if the pain doesn't improve or resolve after a week of analgesia and rest, an MRI should be performed to exclude femoral neck stress fractures. Treatment depends on the location and type of fracture. This diagnosis should be considered when evaluating soldiers and athletes who present with activity-related pain [4].

## Conflict of interest

The authors declare that they have no conflicts of interest concerning this paper.
